# A Noncationic Biocatalytic Nanobiohybrid Platform for Cytosolic Protein Delivery Through Controlled Perturbation of Intracellular Redox Homeostasis

**DOI:** 10.1002/smll.202407676

**Published:** 2024-09-16

**Authors:** Wanyue Lu, Weidong Wang, Yimin Gong, Jianing Li, Yaming Zhou, Yannan Yang

**Affiliations:** ^1^ Shanghai Key Laboratory of Molecular Catalysis and Innovative Materials Department of Chemistry Fudan University Shanghai 200433 China; ^2^ South Australian ImmunoGENomic Cancer Institute The University of Adelaide Adelaide South Australia 5005 Australia; ^3^ Institute of Optoelectronics Fudan University Shanghai 200433 China

**Keywords:** hydrogen‐bonded organic frameworks, intracellular protein delivery, nanobiohybrids

## Abstract

Intracellular delivery of proteins has largely been relying on cationic nanoparticles to induce efficient endosome escape, which, however, poses serious concerns on the inflammatory and cytotoxic effects. Herein, a versatile noncationic nano biohybrid platform is introduced for efficient cytosolic protein delivery by utilizing a nano‐confined biocatalytic reaction. This platform is constructed by co‐immobilizing glucose oxidase (GOx) and the target protein into nanoscale hydrogen‐bonded organic frameworks (HOFs). The biocatalytic reaction of nano‐confined GOx is leveraged to induce controlled perturbation of intracellular redox homeostasis by sustained hydrogen peroxide (H_2_O_2_) production and diminishing the flux of the pentose phosphate pathway (PPP). This in turn induces the endosome escape of nanobiohybrids. Concomitantly, GOx‐mediated hypoxia leads to overexpression of azo reductase that initiated the materials' self‐destruction for releasing target proteins. These biological effects collectively induce highly efficient cytosolic protein delivery. The versatility of this delivery platform is further demonstrated for various types of proteins, different protein loading approaches (in situ immobilization or post‐adsorption), and in multiple cell lines. Finally, the protein delivery efficiency and biosafety are demonstrated in a tumor‐bearing mouse model. This nanohybrid system opens up new avenues for intracellular protein delivery and is expected to be extensively applicable for a broad range of biomolecuels.

## Graphical abstract

A nanobiohybrid platform is developed for intracellular protein delivery. The “self‐destruction” delivery behavior is initiated through the biocatalytic reaction of GOx, which induces the controlled disturbance of intracellular redox homeostasis and triggers the endosomal escape of the biohybrid nanocarriers. The versatile platform shows high delivery efficiency for various types of proteins to different cell lines and in mouse models.

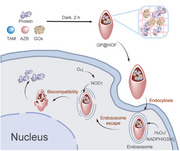

## 1 Introduction

The regulation of proteins is closely linked to human disease.^[^ ^1^ ^]^ Although current research progress shows that intracellular targets may have better effects, the protein drugs in clinics mainly focus on extracellular targets, due primarily to the difficulties in crossing the cell membrane and overcoming endosome entrapment.^[^ ^2^, ^3^, ^4^ ^]^ In recent years, intracellular protein delivery systems, particularly those suitable for in vivo settings, are mostly based on cationic materials including liposomes,^[^ ^5^, ^6^, ^7^ ^]^ polymers^[^ ^8^, ^9^ ^]^ and inorganic nanoparticles.^[^ ^10^, ^11^ ^]^ Although promising, these cationic nanoparticles posed several limitations, including high inflammatory and cytotoxic adverse events.^[^ ^12^ ^]^ These limitations were due primarily to the high cationic charge density of nanoparticles required for inducing efficient endosome escape.^[^ ^13^ ^]^ While surface shielding strategies, e.g., PEGylation,^[^ ^14^ ^]^ can transiently mitigate the biosafety risks, the long‐term risks are still inevitable. How to develop disruptive delivery platforms to bypass the conventional cation‐mediated endosome escape and cytosolic internalization remains a major challenge for safe and efficient protein delivery.

Nanobiohybrids that integrate functional nanomaterials with living systems has become an exciting branch of research at the intersection of materials engineering and biological science. Nanobiohybrids endow organisms with new or enhanced exogenous properties to achieve more powerful biological functions that show promise in fields such as biocatalysis, biosensing, medicine, and robotics.^[^ ^15^ ^]^ For example, the metal–organic framework and the metal‐phenolic network as the hybrid framework have been used to incorporate enzymes through in situ or post‐infiltration preserving enzyme activity and improving catalytic performance.^[^ ^16^, ^17^, ^18^ ^]^ Ge Jun's group developed an encapsulation method for enzymes in amorphous MOFs (aMOFs), which increases the activity of encapsulated GOx by 20 times compared to crystalline MOFs and enables non‐invasive glucose measurement in single living cells to differentiate cancerous from normal cells.^[^ ^19^ ^]^ Hydrogen‐bonded organic frameworks (HOFs) are a class of crystalline porous materials, which are composed of organic building units connected by hydrogen bonds.^[^ ^20^, ^21^ ^]^ Compared to current nanobiohybrids, HOFs containing no metal ions arise much less biosafety concerns, which, together with structural diversity and mild synthesis conditions makes HOFs promising for next‐generation nanobiohybrid materials in biological applications.^[^ ^22^, ^23^ ^]^

**Scheme 1:**
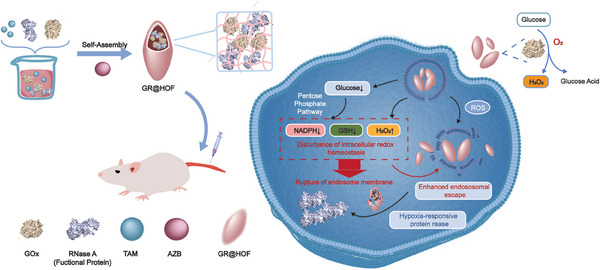
Construction of enzyme‐based nanocomposite and its mechanism of endosomal escape. Synthetic route and schematic illustration of G@HOF‐based Nanobiohybrid Platform for intracellular delivery of functional protein.

In the last years, the amidinium⋯carboxylate interaction has emerged as a powerful tool for the construction of families of three HOFs. These frameworks can be prepared in water and are surprisingly stable, including to heating in polar organic solvents and water.^[^ ^22^ ^]^ Therefore, they are quickly applied to protein encapsulation. However, due to the lack of favorable endosomal escape mechanisms and on‐demand release profile, the nano‐bio hybrid materials reported to date mainly focus on biocatalysis and robotics,^[^ ^24^ ^]^ while their potential application in intracellular delivery of functional proteins have rarely been explored. Here, we introduce a versatile noncationic nano biohybrid delivery platform to realize cytosolic protein delivery. This nanobiohybrid, denoted as G@HOF, was constructed through co‐immobilization of glucose oxidase (GOx)^[^ ^25^, ^26^ ^]^ and the target protein within nanoscale hydrogen‐bonded organic frameworks. The catalytic kinetics of GOx within HOFs was decelerated, resulting in a controlled perturbation of intracellular redox homeostasis through two biochemical effects: 1) sustained hydrogen peroxide (H_2_O_2_) production, and 2) glucose depletion induced decreased flux of the pentose phosphate pathway (PPP),^[^ ^27^, ^28^, ^29^ ^]^ leading to the down‐regulation of triphosphopyridine nucleotide (NADPH) and glutathione (GSH) expression. These two effects cooperatively elevated intracellular oxidative stress in a manner that is sufficient to induce endosome escape while not causing substantial harm to cell viability. Concurrently, GOx‐mediated O_2_ consumption led to intracellular overexpression of azo reductase, initiating self‐destruction of the HOFs by breaking azo bonds and the subsequent release of target proteins.^[^ ^30^, ^31^, ^32^ ^]^ We demonstrated the versatility of this platform for delivering various proteins, different protein loading approaches (in situ immobilization or post‐adsorption), and multiple cell lines. Furthermore, as a proof of concept, the delivery performance of this nano biohybrid platform for a cytotoxic protein was validated in a tumor‐bearing mouse model(**Scheme** **1**).

## 2 Results

### 2.1 Construction of Nanobiohybrid‐Based Delivery Platform

**Figure 1:**
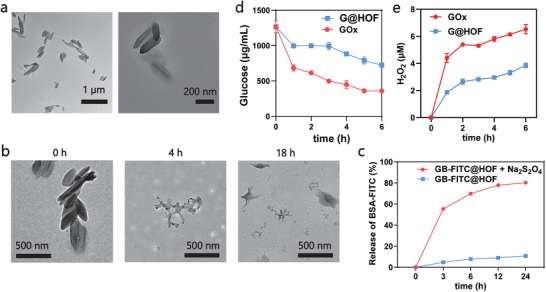
Characterization and catalytic activity of G@HOF. a,b) TEM of HOF, G@HOF and before and after Na_2_S_2_O_4_ treatment (20 mm) with G@HOF for different times. c) BSA‐FITC release from GB‐FITC@HOF in PBS (pH=7.4) with or without Na_2_S_2_O_4_ (20 mm). d) Glucose solutions (1 mg mL^−1^) incubation with free GOx and G@HOF for different times (n=3). e) H_2_O_2_ concentration of glucose solutions (1 mg mL^−1^) incubation with free GOx and G@HOF for different times (n=3).

To realize optimal protein delivery efficiency, we designed GOx‐hybridized nanoscale HOF with controlled catalytic activity to mediate the endosomal escape and intracellular release of protein, as well as bio‐responsive self‐destruction property. The nanoscale HOF was synthesized based on the hydrogen bonding interaction between tetrakis(4‐amidiniumphenyl)methane (TAM) and azobenzenedicarboxylate (AZB) as reported previously,^[^ ^33^, ^34^ ^]^ and GOx was encapsulated in situ to obtain the nanobiohybrid (denoted G@HOF). As characterized by transmission electron microscopy (TEM), HOF and G@HOF showed similar rod‐like morphology with a length of 200 nm and a width of ≈30 nm (**Figure** **1**a; Figure S1 and S23, Supporting Information). The dynamic light scattering (DLS) showed the higher hydrodynamic diameters of G@HOF, which was ≈391 nm (Figure S2, Supporting information). The GOx content was measured to be 88.5 µg mg^−1^ using the Bicinchoninic Acid (BCA) approach. The Fourier‐Transformed Infrared (FTIR) spectrum of suggested the successful synthesis of HOF. Owing to the formation of a hydrogen‐bonded rigid structure, the stretching movement of C═O (1691 cm^−1^) and C═N (1681 cm^−1^) were restricted (Figure S3, Supporting Information). After loading GOx, the zeta potential of the HOF material increased from −23.9 to −18.3 mV, confirming the success of the synthesis of G@HOF. If GOx and RNase A were simultaneously loaded, and the loading capacity was shown in Table S1 (Supporting Information). After protein encapsulation, the zeta potential increased to + 9.8 mV (Figure S4, Supporting Information). G@HOF maintained its rod‐like structure in water for 7 days, and nearly 90% of the proteins were still packaged in nanobiohybrids after 7 days (Figure S7, Supporting information), indicating high water stability. We used EDX mapping to demonstrate that proteins are encapsulated inside the HOF rather than adsorbed on the surface. Cytochrome C (Cyt c) was selected as the model protein and GC@HOF was prepared for iron in Cyt c (C_42_H_52_FeN_8_O_6_S_2_), and it can be recognized and detected by instruments. Through line scanning, we determined the distribution of elements on the GC@HOF surface. As shown in Figure S24 (Supporting Information), the slight distribution of iron indicates that only a little amount of protein was adsorbed on the GC@HOF surface, whereas most of the protein was enclosed in the interior of HOF.

It is known that the azobenzene group could be reduced under hypoxic conditions by the various reductases that are over‐expressed in cells, especially tumor cells. The catalytic process of GOx consumes oxygen and thus provides a hypoxic environment for cells, resulting in intracellular expression of reductases for cleaving the azo bond in G@HOF, which initiates the self‐destruction of the nanohybrid. To verify this self‐destruction behavior, Na_2_S_2_O_4_ (20 mm) was utilized as the reducing agent that simulated the reductase expressed under hypoxia in cells.^[^ ^35^, ^36^, ^37^ ^]^ TEM and scanning electron microscopy (SEM) images showed that the structure of HOFs started to degrade after incubating with Na_2_S_2_O_4_ for 4 h as the rod‐like structure was largely collapsed (Figure 1b). After incubation for 18 h, the structure of HOFs was almost completely destroyed, leaving a large number of debris. The change of morphology during degradation was also characterized by SEM (Figure S5, Supporting information), which revealed the collapse and aggregation of HOFs. These results demonstrated the effect of reductive environment for inducing destruction of the framework. To test whether the self‐destruction would lead to the release of cargo protein, fluorescein 5‐isothiocyanate labeled bovine serum albumin (BSA‐FITC) was used as a model protein to be encapsulated in the G@HOF framework (denoted GB‐FITC@HOF). As shown in Figure 1c, over 80% of BSA‐FITC was released from the nanobiohybrid framework under the simulated reductive conditions (20 mm of Na_2_S_2_O_4_) after 24 h. In contrast, less than 20% of the protein was released at the same time point in the normal condition, suggesting a reductive‐responsive release profile. It is worth noting that, as shown in circular dichroism (CD, Figure S6, Supporting information), the secondary structure of BSA did not change significantly, which proved its integrity. At the same time, the peaks were weakened at 208 and 220 nm after encapsulation and degradation, demonstrating a reductive content of the α‐helical structure of BSA, possibly due to the hydrogen‐bond interaction between its residues and the building blocks of HOFs.

To verify the feasibility of this degradation pathway in cells, we measured the expression of azo reductase after co‐incubation with G@HOF under normoxia and hypoxia conditions, respectively (Figure S8, Supporting Information). After 24 h co‐incubation, the intracellular azo reductase content in G@HOF group was increased, especially in the hypoxia environment, where the expression of azo reductase was up‐regulated by 30.17% compared to the control group, which only increased the expression of azo reductase by 20.62%. These results suggest that G@HOF could induce a reducing microenvironment at the cellular level, which is crucial to promote self‐destruction.

The excessive H_2_O_2_ produced by GOx would potentially pose serious biosafety concerns. Therefore, we tested whether the catalytic kinetics of GOx could be decelerated upon encapsulating in HOF and reduce the H_2_O_2_ production. As shown in Figure 1d,e, the reaction kinetics study show that the rates of glucose consumption and H_2_O_2_ production of G@HOF were significantly reduced to 43.0% and 59.1% compared to that of free GOx, respectively. This considerably decelerated enzyme catalytic kinetics can be attributed to the steric hindrance effect of HOF that limits the contact between substrates and GOx, which would potentially mitigate the cytotoxicity and biological risks caused by excessive H_2_O_2_.

### 2.2 Endosomal Escape Ability and Cytotoxicity of Nanobiohybrid‐Based Delivery Platform

**Figure 2:**
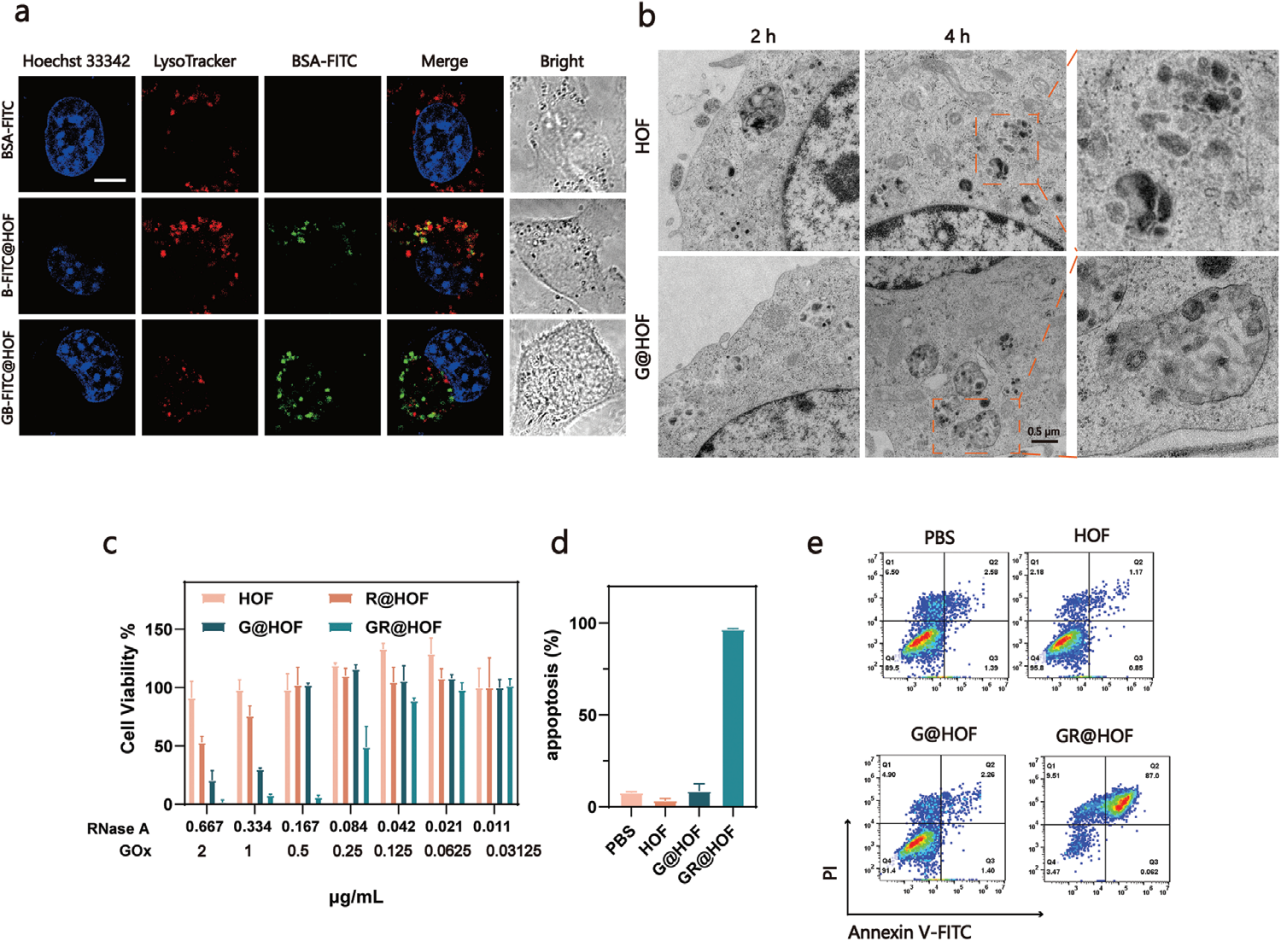
The ability of endosomal escape and protein release of G@HOF‐based nano platform. a) Confocal images exhibiting the endosomal escape of BSA‐FITC, B@HOF, and GB@HOF (10 mg mL^−1^) nanoparticles in 4T1 cells; the lysosomes were stained with Lyso‐Tracker Red probe, shown in red; and the nucleus was stained with Hoechst, shown in blue; nanoparticles was shown in green. Scale bar, 10 µm. b) Bio‐TEM images of HOF and G@HOF after incubation with 4T1 cells for 2 and 4 h, illustrating the endosomal escape of the G@HOF. Scale bar: 0.5 µm. c) Cytotoxicity studies of 4T1cells cultivating with HOF, R@HOF, G@HOF and GR@HOF with low concentration of glucose (0.1 mg mL^−1^) for 24 h. d,e) FCM and quantitative apoptosis analysis of 4T1 cells after difference treatments for 6 h.

The capability of G@HOF to achieve endosomal escape and deliver protein cargoes into the cytoplasm was then evaluated in vitro. As shown in **Figure** **2**a, after incubating 4T1cells with free BSA‐FITC, B‐FITC @HOF (BSA‐FITC encapsulated by HOFs), and GB‐FITC @HOF for 4 h, we detected no apparent co‐localization of FITC and LysoTracker in GB‐FITC @HOF group, suggesting that the endosome membrane was ruptured by GB‐FITC @HOF, and the protein cargoes were released into cytoplasm consequently. On the contrary, due to the lack of GOx‐mediated biocatalytic reaction, B‐FITC @HOF nanoparticles were trapped in the endosomes, leading to co‐localization of FITC and LysoTracker. Additionally, the free BSA‐FITC group showed negligible fluorescence in the cytoplasm due to their cell membrane impermeability. After calculation, the Pearson's correlation coefficient of GB‐FITC@HOF reached at 0.09 while that of B‐FITC @HOF was 0.39, which indicates lower co‐localization of nanoparticle and lysosome in the case of GB‐FITC@HOF, suggesting an efficient endosome escape. The endosome escape behavior was further confirmed by Bio‐TEM (Figure 2b). After incubating HOF with 4T1 cells for 4 h, HOF was still entrapped by endosomes. In contrast, G@HOF nanoparticles diffused outside of endosomes, suggesting that GOx within G@HOF can drive endosomal escape. It is worth mentioning that, the edge of the endosome membrane around G@HOF was unclear, which directly proved the rupture of the endosome membrane. In addition, we observed the residual fragments of G@HOF in the endosomal vesicles, demonstrating the hypoxia‐responsive degradation (Figure S9, Supporting Information).

After verifying the endosomal escape performance of G@HOF, we next sought to test the efficiency of this platform for protein delivery. RNase A is a ribonuclease with potent cytotoxicity, which can degrade a variety of RNA molecules in tumor cells and eventually cause cell apoptosis.^[^ ^38^ ^]^ To visually demonstrate the effect of protein delivery, Western Blot (WB) was used to examine the level of intracellular RNase A (Figure S13a, Supporting Information). It could be determined that after co‐incubation with GR@HOF, the content of intracellular RNase A was increased significantly, suggesting successful intracellular delivery of RNase A. To maintain the biological function of RNase A, they need to be delivered to the cytosol. The anti‐cancer efficacy of various HOFs in 4T1 cells was evaluated using the methyl thiazolyl tetrazolium (MTT) assay. As shown in Figure 2c, RNase A encapsulated by HOF (denote R@HOF) showed over 50% cell viability even at the highest dosage, which is likely due to limited endosomal escape and protein release efficiency. However, GR@HOF showed an extremely high potency in cell inhibition, reducing the 4T1 cell viability to 8% at the dose above 0.167 µg mL^−1^. Flow cytometry analysis further confirmed that the cells apoptosis rate increased to 96.69% for the GR@HOF (Figure 2d,e). In contrast, negligible apoptosis was observed in the other groups. We further employed Calcein‐AM/propidium iodide (PI) double stain assay to confirm the anticancer effect of GR@HOF. The portion of live cells (green) decreased considerably, while that of dead cells (red) largely increased for the cells treated with GR‐HOF, which was consistent with the results of the MTT and apoptosis assays (Figure S10, Supporting Information).

### 2.3 Disruption of Redox Homeostasis by Double Oxidation

**Figure 3:**
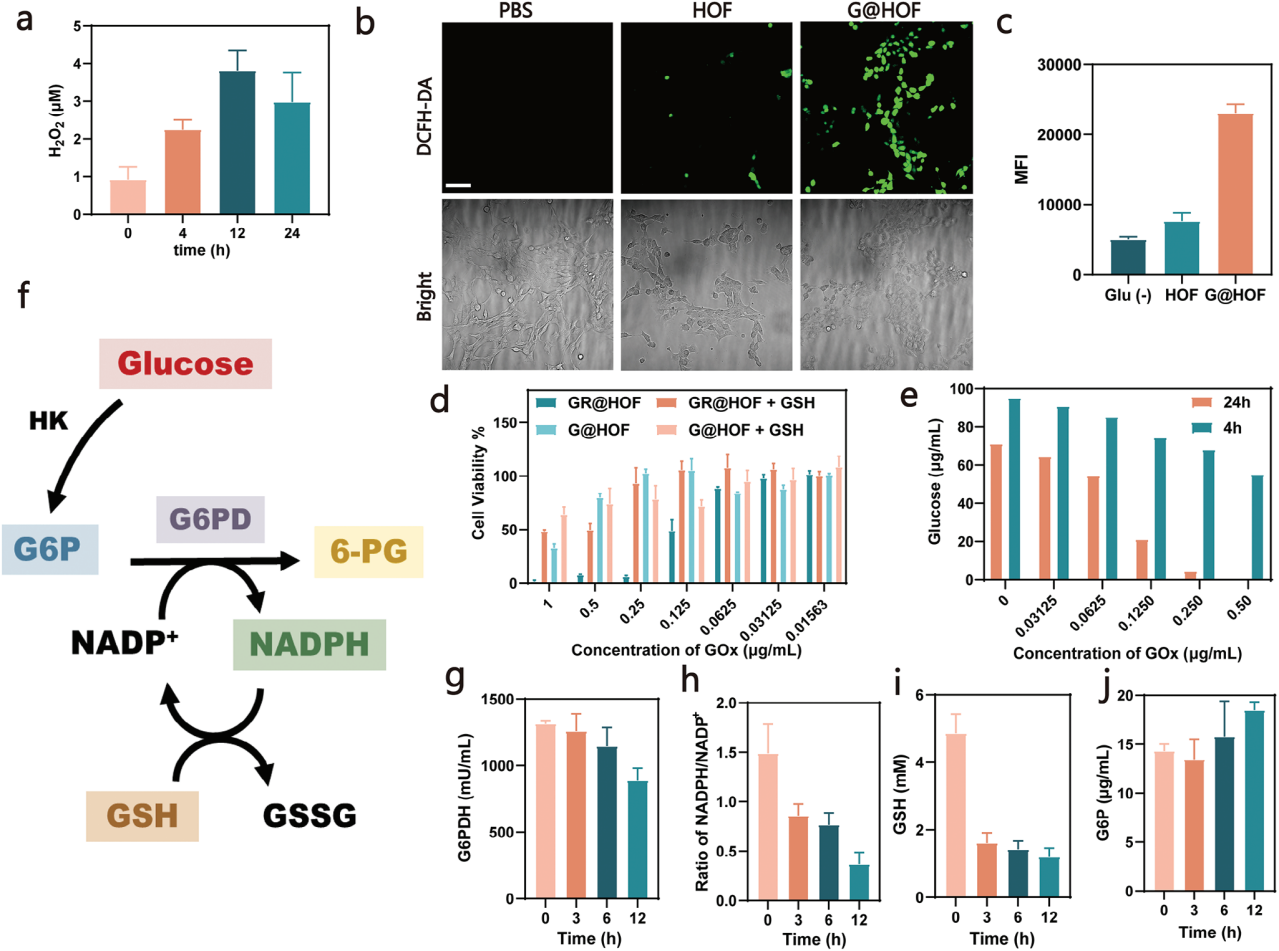
Disruption of redox homeostasis by double oxidation. a) Concentration of intracellular H_2_O_2_ after treatment with G@HOF for 0, 4, 12, 24 h. b) Confocal images of intracellular ROS level in 4T1 cells after 2 h treatment of low glucose, HOF or G@HOF. Scale bar, 100 µm. c) Mean fluorescence intensity (MFI) of DCFH‐DA in 4T1 cells after 2 h treatment of low glucose, HOF or G@HOF. d) Cytotoxicity studies of 4T1cells cultivating with GR@HOF with low concentration of glucose (0.1 mg mL^−1^) with or without GSH (10 mm) for 24 h. e) Concentration of intracellular glucose solution after incubation with G@HOF for 4 or 24 h. f) Schematic diagram of partial Pentose phosphate pathway (PPP). g) The enzymatic activity of G6PDH after treatment with G@HOF for 0, 3, 6, 12 h., unit mU mL^−1^. h) ratio of NADPH/NADP^+^ after treatment with G@HOF for 0, 3, 6, or 12 h. i,j) Concentration of **i** GSH and j) G6P after treatment with G@HOF for 0, 3, 6, or 12 h.

The successful delivery of RNase A has stimulated our interest in exploring the endosomal escape mechanism of G@HOF‐based delivery platform. We first monitored the intracellular H_2_O_2_ level in a time‐dependent manner. After treating 4T1 cells with G@HOF, the intracellular H_2_O_2_ level increased and reached the peak (3.85 µm) at 12 h (**Figure** **3**a). The increased intracellular oxidative stress was further confirmed by strong green fluorescent signals of 2′,7′‐dichlorodihydrofluorescein diacetate (DCFH‐DA) (Figure 3b,c). Next, we explored the effects of this oxidative stress by glutathione (GSH, an intracellular antioxidant) as an H_2_O_2_ neutralizer. First, we verified the neutralization effect of GSH on H_2_O_2_. When incubated with H_2_O_2_, cell viability decreased to 90.8% (Figure S11, Supporting Information). By adding GSH to the medium, the cell viability increased by 104.5%, which proved that GSH could neutralize the toxicity of H_2_O_2_ to 4T1 cells. Accordingly, the cytotoxicity of GR@HOF was largely reduced (Figure 3d) with the addition of GSH, suggesting that the GOx‐mediated oxidative stress played a vital role in inducing endosomal escape. In order to further investigate the reason for the enhancement of cell viability, we compared the effects of G@HOF on cell viability of 4T1 before and after the addition of GSH (Figure 3d). GSH supplementation had no significant effect on rescuing the cells at the tested range of concentrations compared to G@HOF. These results showed that the improvement of cell viability after GR@HOF + GSH treatment compared to GR@HOF was due to the inhibition of endosomal escape, rather than the neutralization of H_2_O_2_. It is worth noting that after adding H_2_O_2_ with the concentration that was equivalent to Figure 3a to the medium, the cell viability remained above 90%. This suggests that G@HOF‐induced oxidative stress does not cause physical damage to cells.

We further examined the variation of glucose concentration during co‐incubation with G@HOF. The results showed that the level of glucose in the medium decreased significantly with the increase of GOx concentration (Figure 3e). In particular, the glucose in the high‐concentration G@HOF group was almost completely depleted after 24 h, while the cells in the control group only consumed ≈30% of the glucose in the medium at the same time point.

The pentose phosphate pathway (PPP) is a major pathway for glucose catabolism, playing a vital role in regulating cell growth by supplying cells with NADPH for detoxification of intracellular and maintaining intracellular redox homeostasis (Figure 3f). Upon entering cells through a glucose transporter, glucose is phosphorylated by hexokinase to form glucose‐6‐phosphate (G6P). G6P can be dehydrogenated by glucose‐6‐phosphate dehydrogenase (G6PDH), which was then converted to 6‐phosphoglucono‐δ‐lactone (6‐PG). This irreversible reaction can generate the reducing agent NADPH, which regulates intracellular redox homeostasis by reducing oxidized glutathione (GSSG) to GSH to remove excess ROS in cells.^[^ ^39^, ^40^, ^41^ ^]^ We assumed that the exogenous consumption of glucose by G@HOF would directly affect the flux of PPP, break the redox balance in cells, aggravate oxidative stress, and induce endosomal damage.

In order to evaluate the effect of G@HOF on PPP flux, we examined the enzymatic catalytic activity of G6PDH (Figure 3g) and the expression levels of related molecules (G6P, NADPH, and GSH) (Figure 3h–j) by treating cells with G@HOF. As shown in Figure 3g, the activity of G6PDH decreased to 887 mU mL^−1^ after G@HOF co‐incubation for 12 h. The decreased G6PDH in turn caused a decrease of PPP flux and consequently affected the intracellular redox homeostasis. As expected, the ratio of NADPH/NADP^+^ was significantly down‐regulated after 12 h, which proved that the catalytic activity of G6PDH was inhibited and the original redox balance was broken. The level of GSH decreased to 24% of the initial time, further confirming the imbalance of redox homeostasis. However, the level of active intracellular G6P continued to increase within 12 h. This might be due to the disruption of redox homeostasis that promoted cells to uptake more of glucose to produce G6P.

In order to further verify the role of PPP inhibition‐mediated endosome rupture, we added different amounts of G6P to the medium and examined the cytotoxicity of GR@HOF. Exogenous G6P provided a sufficient initial substrate for PPP, which would help cells restore the activity of the pathway and regulate intracellular redox balance. As shown in Figure S12 (Supporting Information), when the concentration of G6P was 2000 µg mL^−1^, the cell viability was restored to 18%, which proved that the normalization of PPP flux would partially restore the redox homeostasis, thus reducing the level of intracellular oxidative stress and the rupture of endosomes (Figure S12, Supporting Information). The above observations suggested an endosomal escape mechanism based on perturbation of intracellular redox homeostasis. When G@HOF was endocytosed into endosomal vesicles, GOx mediated excessive H_2_O_2_ production in cells, ruptured the endosomal membrane, caused intracellular oxidative stress, and induced endosomal escape. At the same time, the catalytic action of GOx caused abnormal glucose consumption, and reduced the flux of the PPP, which is one of the glucose metabolic pathways. These two down‐regulated the expression of downstream NADPH and reduced the production of intracellular reducing molecular (GSH), and finally disturbed the cellular redox homeostasis, weakened the cellular antioxidant defense mechanism, and finally aggravated oxidative stress and endosomal escape.

### 2.4 Evaluation of the Versatility of the Protein Delivery Platform

**Figure 4:**
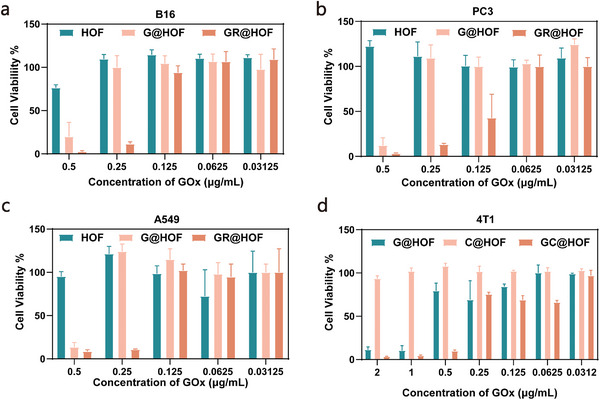
Evaluation of versatility of delivery platform. a–c) Cytotoxicity studies of a) B16F10, b) PC3 and c) A549 cells cultivating with HOF, G@HOF and GR@HOF for 24 h. d) Cytotoxicity studies of 4T1 cells cultivating with G@HOF, C@HOF and GC@HOF for 24 h.

We evaluated the performance of G@HOF‐based delivery platform in different multiple cell lines, including B16F10, PC3, and A549 cell lines. As shown in **Figure** **4**a, in B16F10 cells, at the GOx concentration of 0.25 µg mL^−1^, the cell viability of G@HOF was 99.92%, while the cell viability after GR@HOF treatment was only 11.39%, which suggested the high efficiency of RNase A delivery in B16F10 cells, and evidenced that decreased of cell viability was due to the RNase A mediated degradation of cytoplasmic RNA rather than GOx mediated ROS production. Similar results were obtained in PC3 and A549 cells (Figure 4b,c). GR@HOF could effectively reduce the viability of corresponding cells at 0.25 µg mL^−1^ GOx concentration, which indicated the versatility of the G@HOF delivery platform for different cell lines. To further understand the steep drop of cell viability from 100% to ≈20%, the increment of concentration in the experiment is reduced to 0.0125 µg mL^−1^ for B16F10, A549 and PC‐3 cells and 0.0375 µg mL^−1^ for 4T1 cells. It can be determined that ≈0.1625 and 0.2375 µg mL^−1^, the cell viability reached to the cutoff point of concentration for cytotoxicity in different cell lines (Figure S22, Supporting Information).

Next, we demonstrated the capability of the G@HOF delivery platform for other functional proteins with different surface charges and molecular weights. Cytochrome c (Cyt c) is another well‐known cytotoxic protein, that playing an important role in the process of apoptosis.^[^ ^42^, ^43^ ^]^ We synthesized Cyt c encapsulated G@HOF (denoted GC@HOF) and examined the cell‐killing effect of GC@HOF on 4T1 cells by MTT assay (Figure 4d). Compared with G@HOF and C@HOF, GC@HOF decreased the cell viability sharply to 9.82% after co‐incubated with 4T1 for 24 h. Furthermore, WB was used to detect the level of intracellular Cyt c as well (Figure S13b Supporting information). The results showed that with the incubation of GC@HOF, a higher level of Cyt c could be detected, indicating the successful cytosolic delivery and well‐maintained biological function of Cyt c through the G@HOF delivery platform. In addition, G@HOF platform can also encapsulate other proteins, including β‐galactosidase (β‐Gal) (denoted Gβ@HOF) and BSA‐FITC (denoted GB@HOF) with high content (Table S1, Supporting Information), and the morphology and size of the Gβ@HOF and GB@HOF were well‐maintained (Figures S14–S16, Supporting Information), suggesting that the G@HOF delivery platform had good compatibility with proteins with different molecular weights and surface charges.

In order to expand the adaptability of the G@HOF platform to various proteins, we tested the feasibility of using a post‐adsorption approach to load the target functional proteins (RNase A, β‐Gal, Cyt c, and BSA‐FITC) and formed G@HOF‐RA, G@HOF‐βGal, G@HOF‐Cytc, and G@HOF‐B. The loading capacity of functional proteins is shown in Table S1 (Supporting Information). We found that the loading capacity of functional proteins obtained by the post‐adsorption approach was lower than that by in situ encapsulation. For example, after the post‐adsorption approach, the loading capacity of RNase A was reduced to 3.5%. Some reports demonstrated that protein residues will contribute to forming hydrogen‐bonded connections, which might explain this decrease in loading capacity for the proteins participating in the self‐assembly process of HOFs.^[^ ^33^, ^44^ ^]^ The results of the cell viability test showed that G@HOF‐RA and G@HOF‐Cytc showed higher cytotoxicity than G@HOF at the same concentration of GOx (Figure S15, Supporting Information), which demonstrated that G@HOF can also achieve efficient delivery for post‐adsorbed proteins. In general, we demonstrated the versatility of G@HOF platform from three dimensions: application in different cell types, protein types, and protein loading method as well. Therefore, the G@HOF delivery platform can develop personalized delivery systems for different cells and different functional proteins, providing a powerful tool for intracellular delivery of therapeutic proteins, without considering the additional effect such as molecular weight and surface charges of proteins for intracellular delivery. In general, low molecular weight and positively charged proteins will obtain higher loading efficiency in the system, which will facilitate better delivery efficiency.

### 2.5 In Vivo Pharmacokinetics and Biodistribution

We next attempted to test the performance of the delivery platform in vivo. To improve the suitability for in vivo application, we modified GR@HOF with hyaluronic acid (HA). As shown in (Figure S17, Supporting Information), the surface modification did not change the morphology of the nanoparticles. After modification, the zeta potential decreased from + 9.2 to −13.4 mV (Figure S18, Supporting Information), which proved the successful synthesis of hyaluronic acid‐modified GR@HOF (GR@HOF‐HA). We also prepared HA modified G@HOF (G@HOF‐HA) and R@HOF (R@HOF‐HA) for comparison. HA was used as part of a delivery system to extend the blood circulation time of GR@HOF nanobiohybrids and enhance therapeutic efficacy in vivo. More importantly, HA has an affinity with the CD44 receptor and can be used as a tumor‐targeting ligand, which can functionalize GR@HOF with tumor selectivity. After surface modification, we evaluated the biosafety of G@HOF‐HA and GR@HOF‐HA in normal Cells using Human Umbilical Vein Endothelial Cells (HUVEC) (Figure S19, Supporting information). The results show that G@HOF‐HA and GR@HOF‐HA showed cytotoxicity only at the maximum concentration of 200 µg mL^−1^. On the other hand, the cell viability of 4T1 cells decreased to 11.04% at the same concentration. Therefore, it was proved that after coating with HA, the nanoplatform has no obvious killing effect on normal cells, but has tumor targeting.

**Figure 5:**
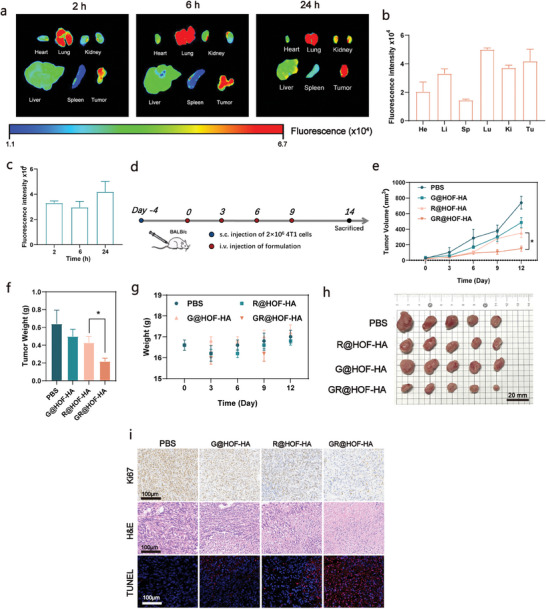
In Vivo biodistribution and antitumor efficacy. a) Representative photographs and bioluminescence images of organs ex vivo after euthanasia show the targeting of GR@HOF‐HA toward tumor. b) Quantifications of fluorescence intensity of heart, liver, spleen, lung, kidney and tumor after intravenous injection for 24 h (n=3). c) Quantifications of fluorescence intensity intensity of tumor after intravenous injection for 2, 6, 24 h (n=3). d) Experimental outline showing the treatment steps and procedures for evaluating the therapeutic outcomes of different groups in 4T1 tumor‐bearing Balb/c mice. e) Average tumor growth curves of the 4T1 tumor‐bearing Balb/c mice after various treatments and f) gross tumor images and g) corresponding tumor weight after 14 days. The mice treated with PBS were set as the control group. Data are presented as mean ±  SEM. n=5 mice per group. h) Photograph of tumors peeled from mice at the end of treatment. i) Ki67, H&E and TUNEL‐stained images of the dissected tumor tissues after 14 days of treatment.

The pharmacokinetic properties of GR@HOF‐HA were evaluated in Balb/c mice following intravenous injection. The GR@HOF‐HA concentration‐time curve is illustrated in Figure S20 (Supporting Information). According to the calculated major pharmacokinetic parameters (Table S2, Supporting Information), the t_1/2_ of GR@HOF‐HA was 5.097 h. The biodistribution and tumor accumulation of GR@HOF‐HA were observed using a fluorescence intensity imaging system. After intravenous injection of GR@HOF‐HA, the time course of the FL signal variation was monitored in vivo. The fluorescence intensity of GR@HOF‐HA at the tumor sites peaked at 24 h post‐injection and showed a significant enhancement compared to that at 0 and 6 h (Figure 5c), implying high tumor accumulation and long retention of GR@HOF‐HA. Tumor tissues and major organs were collected to study the biodistribution profiles. Ex vivo fluorescence images (**Figure** **5**a) and semi‐quantification results (Figure 5b) showed strong fluorescence intensity in the tumors, further confirming that GR@HOF‐HA exhibited high tumor accumulation and long retention.

### 2.6 In Vivo Protein Delivery for Antitumor Efficacy

In view of the good tumor accumulation behavior of GR@HOF‐HA, we tested the antitumor efficacy. A xenograft breast cancer tumor model was established by subcutaneously injecting 4T1 cells in Balb/c mice (Figure 5d). When the tumor volume reached ≈30 mm^3^, the tumor‐bearing mice were randomly divided into 4 groups, and PBS, G@HOF‐HA, R@HOF‐HA, and GR@HOF‐HA were injected intravenously once every 3 days with a total of four times. As shown in Figure 5e,h, the average tumor volume of the PBS group was more than 750 mm^3^ on the 12th day. Compared with other groups, the tumor growth of the GR@HOF‐HA group was significantly inhibited. The average tumor volume was ≈200 mm^3^, and the tumor weight was significantly lower than that of other groups (Figure 5f), confirming the anti‐tumor effect of GR@HOF‐HA. During the treatment period, there was no significant change in the body weight of the mice (Figure 5g). Finally, the main organs and tumor tissues were collected to further study the biosafety and therapeutic effect. Hematoxylin Eosin staining (H&E) and terminal deoxynucleotidyl transferase‐mediated Dutp‐biotin nick end labeling (TUNEL) tumor staining methods were used to observe the morphological changes and apoptosis of tumor tissues. As shown in Figure 5i, GR@HOF‐HA group showed obvious apoptotic morphology, mainly manifested as tumor cell nucleus shrinking, even missing and tumor cell gap increasing. TUNEL staining images further confirmed this result. Its biocompatibility was evaluated in the heart, liver, spleen, lung, and kidney (Figure S21, Supporting Information).

## 3 Conclusion

In this study, we have developed a non‐cationic nanobiohybrids delivery platform for cytoplasmic protein delivery. G@HOF created an hypoxia microenvironment by consuming intracellular oxygen and took advantage of the overexpressed azo reductase under this state to initiate the “self‐destruction” of the carrier and release the protein cargoes. In the process of protein delivery, G@HOF continuously produced H_2_O_2_ through the biocatalytic reaction of GOx and consumed glucose to reduce the flux of the PPP pathway, finally inducing perturbation of intracellular redox homeostasis. This disturbance would promote endosome escape of nanohybrids, while the cells would not die from the oxidative stress during the process. Utilizing G@HOF nanohybrids, we realized the delivery for different proteins, different cell lines in vitro, and tumor‐bearing mice in vivo, demonstrating the high efficiency and versatility of the delivery platform.

For clinical translation, achieving precise and targeted delivery to cancer cells is a crucial research direction. For instance, Liu's group^[^ ^45^ ^]^ has also developed a biodegradable nano‐molecularly imprinted polymer for the targeted delivery of protein drugs using sialic acid as the targeting ligand. In our study, we coated the surface of the nanohybrid system with HA, which is a well‐known ligand for CD44 overexpressed in many types of cancer stem cells. While we did not test the targeting activity of our nanosystem in the present work, we believe that the nanobiohybrid particles can potentially target CD44 overexpressed cancer cells. Moreover, given the existence of reactive chemical groups, such as carboxyl and amines, on the surface of HOF nanoparticles, the conjugation of other types of targeting ligands, for instance, aptamers and antibodies are also possible. This will endow the delivery platform with optimal targeting capability to diverse types of cancer cells, which will be comprehensively explored in our future work.

Compared with the traditional porous framework materials MOF, HOF materials have high loading capacity and safety of non‐metal ions. At the same time, the retention of pore structure is conducive to mass transfer, enabling confined biocatalysis and cytoplasmic delivery. Additionally, our work for the first time demonstrated the promise of GOx as an endosome escape promoter and highlight its critical role in nano biohybrid protein delivery systems, broadening the application of GOx in the field of protein therapy. This work introduces a nanobiohybrid intracellular protein delivery platform and provides proof of concept through in vitro and in vivo validation. We acknowledge that there is still a gap toward practical application, and more implementations are needed in future work to verify the efficiency and safety of its delivery in vivo.

## 4 Experimental Section

### Materials

All chemicals were obtained from Bide Pharmaceuticals. GOx and HA were purchased from Aladdin, while Lyso‐Tracker Red, Glucose Assay Kit, G6PDH Activity Assay Kit, Hydrogen Peroxide Assay Kit, and GSH and GSSG Assay Kit and Western Blot (WB) antibodies were purchased from Beyotime Institute of Biotechnology. Hoechst33342 was purchased from Thermofisher.DCFH‐DA was purchased from Sigma–Aldrich. Methyl thiazolyl tetrazolium (MTT), RNase A, and Cytochrome C were purchased from BBI Life Sciences Limited, FITC Conjugated BSA, paraformaldehyde, PI, and calcein AM was purchased from Solarbio. Paraformaldehyde was purchased from Biosharp. RPMI 1640 and DMEM culture medium were purchased from Genom, and fetal bovine serum (FBS) was purchased from ExCell Bio. All cells were obtained from Fenghui Biotechnology Co., Ltd.

### Characterizations

The morphology and size of HOF, G@HOF, GP@HOF, and nanoparticles after degradation and EDX mapping analysis were studied using a transmission electron microscope (HT7800 TEM) and a scanning electron microscope (Phenom Prox, SEM). The zeta potentials and DLS of HOF, G@HOF, and GP@HOF were measured on a Zetasizer Nano‐ZS size analyzer. The FTIR absorption spectra of HOF, G@HOF, and GP@HOF were measured by a Nicolet iS10 FTIR Spectrometer (Thermofisher). Circular dichroism spectra of free BSA and GB@HOF+Na_2_S_2_O3_4_ were measured by circular dichroism (Applied Photophysics Ltd., Chirascan, CD). The improved chemiluminescence WB detection technique was used to visualize the proteins detected.

### Synthesis of HOF, G@HOF, and GP@HOF

The synthesis of HOF was reported. For the synthesis of G@HOF and GP@HOF (P = RNase A, BSA‐FITC, β‐Gal or cytochrome c), typically, 0.2 mg GOx and 0.4 mg functional protein was added to TAM. After the mixture was stirred at room temperature for 20 min, AZB was added to the mixture under stirring, with the ratio of TAM: AZB = 1: 4, and the volume of the mixture was adjusted to 880 µL by adding water. After stirring for an additional 2 h in the dark, the resulting yellow suspension was centrifugated to afford HOF/G@HOF/GP@HOF. The resulting yellowish product was washed with water three times to remove unreacted precursors. The yellow powder product was obtained by vacuum drying at room temperature overnight.

### Preparation of G@HOF‐HA and GR@HOF‐HA

To improve hydrophilicity, 2 mg G@HOF or GP@HOF dispersed in deionized water containing 4 mg of HA under magnetic stirring overnight in the dark. After centrifugation (12 000 rpm, 30 min), the hydrophilic G@HOF‐HA and GR@HOF‐HA were collected.

### Preparation of GR@HOF‐HA‐Cy5

For the fabrication of GR@HOF‐HA‐Cy5, 5 mg GR@HOF‐HA was dispersed in deionized water containing NaHCO_3_ (0.1 m, 2 mL). After adding Cy5 (1 mm, 125 µL in DMSO), the mixture was stirred overnight in the dark. The resulting green suspension was centrifugated to afford GR@HOF‐HA‐Cy5. The product was washed with ethanol solution (ethanol: water = 1:3).

### Protein Content Assay

The content of protein in GP@HOF or GP@HOF‐HA was determined by the BCA method. The following formulae were used to compute the loading capacity (LC) and loading efficiency (LE) of GOx or protein in GP@HOF.

(1) $$\mathrm{LE}\;\left(\%\right)=\frac{\mathrm{Weight}\;\mathrm{of}\;\mathrm{GOx}\;\mathrm{or}\;\mathrm{protein}\;\mathrm{loaded}\;\mathrm{in}\;\mathrm{GP}@\mathrm{HOF}\;\times 100\%}{\mathrm{Weight}\;\mathrm{of}\;\mathrm{GOx}\;\mathrm{or}\;\mathrm{protein}\;\mathrm{fed}\;\mathrm{initially}}\;$$

(2) $$\mathrm{LC}\;\left(\%\right)=\frac{\mathrm{Weight}\;\mathrm{of}\;\mathrm{GOx}\;\mathrm{or}\;\mathrm{protein}\;\mathrm{loaded}\;\mathrm{in}\;\mathrm{GP}@\mathrm{HOF}\;\times 100\%}{\mathrm{Weight}\;\mathrm{of}\;\mathrm{GP}@\mathrm{HOF}}\;$$

### Hypoxia‐Responsive Protein Release of GB@HOF

To demonstrate the hypoxia‐induced cleavage of azobenzene moieties, BSA‐FITC was encapsulated into G@HOF nanocomposites to form GB@HOF. GB@HOF was dissolved in DI water at 2 mg mL^−1^ and treated with or without Na_2_S_2_O_4_ (final concentration of 20 mm) for 24 h. The morphology of GB@HOF before and after Na_2_S_2_O_4_ treatment was determined by transmission electron microscope. The release of BSA‐FITC was determined by a microplate reader (BioTek Synergy H1) at λ_ex_ = 488 nm and λ_em_ = 525 nm.

### Hypoxia‐Responsive Expression of Azo Reductase of 4T1 Cells

To demonstrate the upregulation of azo reductase in hypoxic conditions, 4T1 cells were seeded in the 6‐well plates at the density of 3 × 10^5^ cells per well. The first group was incubated under standard conditions (containing humidified 5% CO_2_/95% air at 37 °C) for 12 h. The second group was incubated under hypoxic conditions (1% O_2_) for 12 h. After the addition of cell lysis buffer to each group, the mixture was centrifuged at 5000 rpm for 5 min and the supernatant was collected. Then 1 µmol of NADPH, and 8 nmol of orange IIweres added to each group separately. The concentration of azo reductase was spectrophotometrically assayed at room temperature at 513 nm.

### Catalytic Reactivity

To evaluate the catalytic activity of G@HOF in comparison with GOx, G@HOF, and GOx (of the same concentration of GOx) were separately added to glucose solution (1 mg mL^−1^) for 6 h. The pH change during the reaction was monitored with a pH meter. A glucose detection kit and an H_2_O_2_ detection kit were used to determine the content of glucose and H_2_O_2_ in the solution at different time points according to the manufacturer's protocol.

### Cell Culture

4T1 and A549 cells were cultured in RPMI 1640 medium (Genomcell Bio). PC3 and B16F10 cells were cultured in a DMEM medium (Genomcell Bio). All medium was supplemented with 10% fetal bovine serum (Excell), and 1% penicillin streptomycin solution (Sigma–Aldrich). All cells were maintained in a humidified atmosphere containing 5% CO_2_ at 37 °C.

### Cytotoxicity

The MTT assay was used to assess the cytotoxicity of GOx, HOF, G@HOF, GP@HOF, G@HOF‐P, G@HOF‐HA, and GR@HOF‐HA. 96‐well plates were seeded with 8 × 10^3^ 4T1 or B16F10 or PC3 or A549 or HUVEC cells per well and incubated overnight. Different concentrations of materials were dispersed in glucose‐free RPMI 1640 medium (with additional glucose at 100 µg mL^−1^) and added to 96‐well plates. After incubating for 24 h, the original medium was discarded, and glucose‐free medium containing 10% MTT (5 mg mL^−1^) was added to per well and incubated for 4 h. Finally, 100 µL of dimethyl sulfoxide (DMSO) was added to each well to dissolve the formazan crystals. The optical density (OD) was recorded at 570 nm in a microplate reader and the percentage of cell viability was determined.

### Endosomal Escape of GB@HOF

The endosomal escape of GB@HOF containing BSA‐FITC were visualized by confocal laser microscopy (CLSM, Nikon C2+). Briefly, 4T1 cells were seeded in a confocal dish at the density of 10 × 10^4^ cells and were incubated overnight before treatment with BSA‐FITC, B@HOF, or GB@HOF in low‐glucose medium at the FITC‐RPAB concentration of 10 µg mL^−1^ for 6 h. Cells were washed three times with PBS, stained with Lysotracker Red (200 nm) and Hoechst 33342 (5 µg mL^−1^), and observed by CLSM.

### Bio‐TEM of HOF or G@HOF‐Treated 4T1 Cells

The cell uptake of HOF and G@HOF by 4T1 cells was observed with a TEM (JEOL‐1230, JEOL). After incubation with 10 µg mL^−1^ HOF or G@HOF for 6 h respectively, the 4T1 cells were prefixed with 2.5% glutaraldehyde at 4 °C for 4 h. Then the cells were post‐fixed with 1% osmium tetroxide at 4 °C for 1 h. Both fixation and post‐fixation steps included final rinsing in ultra‐pure water and then staining with 0.5% uranyl acetate at 4 °C. The cells were dehydrated through a series of ethyl alcohol concentrations (i.e., 30%, 50%, 70%, 80%, 90%, 100%, and dry alcohol) for 10 min each. Then, the cells were treated with propylene oxide, followed by 1 : 1 propylene oxide : resin for 2 h. The cells were infiltrated in resin at 70 °C for 24 h and ultramicrotomy was conducted. Then, the samples were observed with a TEM at 80 kV.

### Cell Apoptosis

Cell apoptosis of 4T1 cells was examined using the Annexin V‐FITC/PI Apoptosis Detection Kit (Elabscience). 4T1 cells were seeded at 2 × 10^5^ cells per well in six‐well plates and incubated overnight. After discarding the original medium, HOF, G@HOF, and GR@HOF at a concentration of 50 µg mL^−1^ dispersed in low‐glucose medium were added to incubate for another 6 h. Afterward, the cells were collected and stained with annexin V‐FITC and propidium iodide (PI) according to the manufacturer's protocol. Finally, the cells were detected by flow cytometry.

Besides, 4T1 cells were seeded at 1 × 10^5^ cells per dish in confocal glass bottom dishes overnight and were co‐incubated with various nanomaterials (50 µg mL^−1^) for 6 h. At last, the cell necrosis was observed by CLSM, after being stained with calcein‐AM and PI according to the manufacturer's instructions (Solarbio).

### Disruption of Intracellular Redox Homeostasis by Double Oxidation

First, the consumption of glucose was examined. 4T1 cells were seeded at 2 × 10^5^ cells per well in 6‐well plates and incubated overnight. After discarding the original medium, G@HOF at a concentration of 50, 25, 12.5 6.25, 3.125, 0 µg mL^−1^ with dispersed in low‐glucose medium were added to incubate for 4 and 24 h respectively. The concentration of glucose was determined by Glucose Assay Kit with O‐toluidine (Beyotime Biotechnology)

Next, the disruption of redox homeostasis was examined by the expression of key proteins in the PPP pathway. 4T1 cells were seeded at 2 × 10^5^ cells per well in 6‐well plates and incubated overnight. After discarding the original medium, G@HOF at a concentration of 25 µg mL^−1^ dispersed in low‐glucose medium were added to incubate for 0, 3, 6, and 12 h. Then, the Intracellular expression level of G6P, G6PDH, GSH and GSSG and NADP^+^/NADPH was examined by G6P Assay Kit with WST‐8, G6PDH Activity Assay Kit with WST‐8, GSH and GSSG Assay Kit and NADP^+^/NADPH Assay Kit with WST‐8 (Beyotime Biotechnology) according to the manufacturer's protocol.

Besides, the level of intracellular H_2_O_2_ was examined. 4T1 cells were seeded at 2 × 10^5^ cells per well in 6‐well plates and incubated overnight. After discarding the original medium, G@HOF at a concentration of 25 µg mL^−1^ with dispersed in low‐glucose medium were added to incubate for 0, 4, 12, and 24 h. The level of Afterward, the concentration of H_2_O_2_ in different wells was determined by the H_2_O_2_ assay kit (Beyotime Biotechnology). The cytotoxicity of H_2_O_2_ at the concentration of examined level was then tested by co‐incubation of 4T1 and H_2_O_2_ (4 µm) with or without the addition of GSH for 24 h. The MTT assay was utilized to quantify this cytotoxicity.

Finally, the cytotoxicity of G@HOF and GR@HOF was examined after removing double oxidation by MTT assay. 96‐well plates were seeded with 8 × 10^3^ 4T1 cells per well and incubated overnight. Different concentrations of materials were dispersed in glucose‐free RPMI 1640 medium (with additional glucose at 100 µg mL^−1^ and glutathione at 20 mm) and added to 96‐well plates. After incubating for 24 h, the original medium was discarded, and glucose‐free medium containing 10% MTT (5 mg mL^−1^) was added to per well and incubated for 4 h. Finally, 100 µL of DMSO was added to each well to dissolve the formazan crystals. The OD was recorded at 570 nm in a microplate reader and the percentage of cell viability was determined.

### Pharmacokinetics of GR@HOF‐HA‐Cy5 NPs

To evaluate the in vivo pharmacokinetic, Balb/c mice (n = 3) were intravenously injected with GR@HOF‐HA‐Cy5 NPs (GOx concentration of 0.72 mg kg^−1^, 100 µL). At post‐injection of 0.16, 0.5, 1, 2, 6, 12, and 24 h, the blood (20 µL) was collected via the tail vein and added into deionized water at a volume of 80 µL to disperse the NPs. The concentration of GR@HOF‐HA‐Cy5 NPs was obtained by measuring the fluorescence intensity of the blood sample (10 µL blood, 10 µL heparin sodium solution, and 40 µL purified water). A two‐compartment pharmacokinetic model was used to analyze the data and to calculate the pharmacokinetic parameters such as distribution and elimination of half‐lives (Td_1/2_, Te_1/2_) from the percentages of the initial blood concentration C_0_ with Drug and Statistics for Windows 2.0 software (SAS Inc., Cary, NC).

### Biodistribution Study

GR@HOF‐HA‐Cy5 NPs tissue distributions in Balb/c mice were investigated, which administered i.v. (GOx formulations at a dose of 0.72 mg kg^−1^, equivalency). The mice were sacrificed at each time (2, 6, and 24 h), and the major organs (heart, liver, spleen, lung, kidney) and tumor were collected. A maestro in the vivo imaging system was used for further ex vivo fluorescence.

### In vivo Antitumor Experiments

The 4T1 tumor‐bearing Balb/c mice were divided into four groups (n = 5 per group) for different treatments as follows for five times (with an interval between each injection of 2 day): 1) i.v. Injected with PBS (control), 2) i.v. Injected with G@HOF‐HA solution, 3) i.v. injected with R@HOF‐HA suspension, and 4) i.v. injected with GR@HOF‐HA suspension. Note that groups 2 and 4 had the same GOx dose of 0.6 mg kg^−1^, and groups 3 and 4 had the same RNase A dose of 0.2 mg kg^−1^. All of the injection volumes were fixed at 100 µL. The tumor size and body weight were recorded for 14 d. Tumor volume (*V*) was calculated as width^2^ × length/2. 14 days after the drug administration, the mice were sacrificed, and major organs (lung, heart, liver, kidney, and spleen) were used for the tissue slides and histopathological assessment. The isolated tumors were weighted and imaged.

## Conflict of Interest

The authors declare no conflict of interest.

## Supporting information



Supporting Information

## Data Availability

The data that support the findings of this study are available from the corresponding author upon reasonable request.
