# Supplementary material for: A Noncationic Biocatalytic Nanobiohybrid Platform for Cytosolic Protein Delivery Through Controlled Perturbation of Intracellular Redox Homeostasis
Source: Small. 2024 Sep 16;20(49):2407676. doi: 10.1002/smll.202407676 (PMC11618714; doi:10.1002/smll.202407676)
Supplement: Supplementary file 1 — Supporting Information [file SMLL-20-2407676-s001.docx]

Supporting Information of

**A Noncationic** **Biocatalytic Nanobiohybrid Platform for Cytosolic Protein Delivery through Controlled Perturbation of Intracellular Redox Homeostasis**

*Wanyue Lu, Weidong Wang, Yimin Gong, Jianing Li, Yaming Zhou* and Yannan Yang**

W. Lu., W. Wang., Y. Gong, J. Li, Y. Zhou

Shanghai Key Laboratory of Molecular Catalysis and Innovative Materials, Department of Chemistry, Fudan University, Shanghai, 200433, China

Email: ymzhou@fudan.edu.cn

Y. Yang

South Australian immunoGENomic Institute, Faculty of Health and Medical Sciences, The University of Adelaide, Adelaide, South Australia, 5005, Australia

Email: yannan.yang@adelaide.edu.cn

Y. Yang

Institute of Optoelectronics, Fudan University, Shanghai, 200433, China


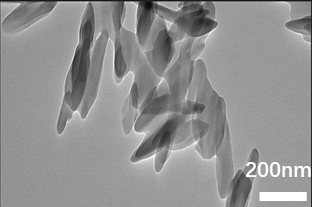


**Figure S1. TEM image of HOF.**

**Figure S2. DLS result of G@HOF.**


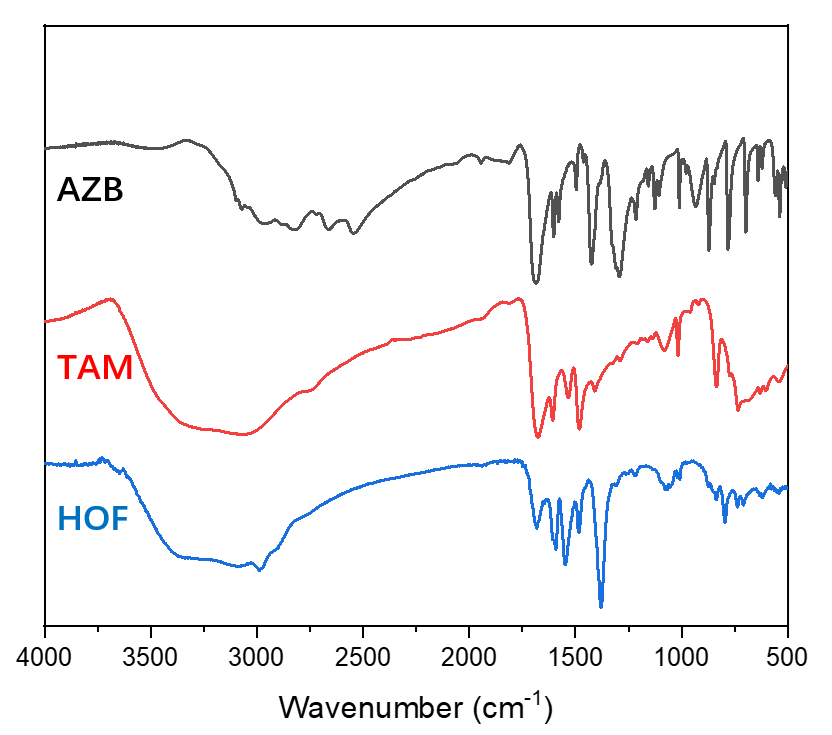


**Figure S3. FTIR spectrum of TAM, AZB and HOF.**

**Figure S4. Zeta potential of HOF, G@HOF, GR@HOF.**

**(a)**


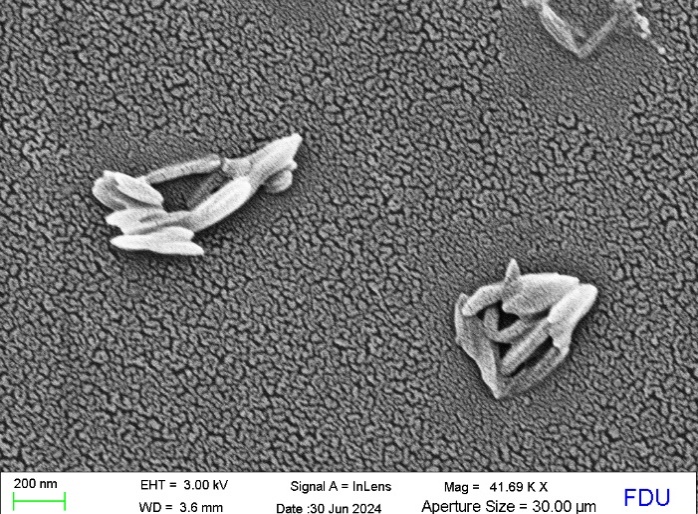


**(b)**


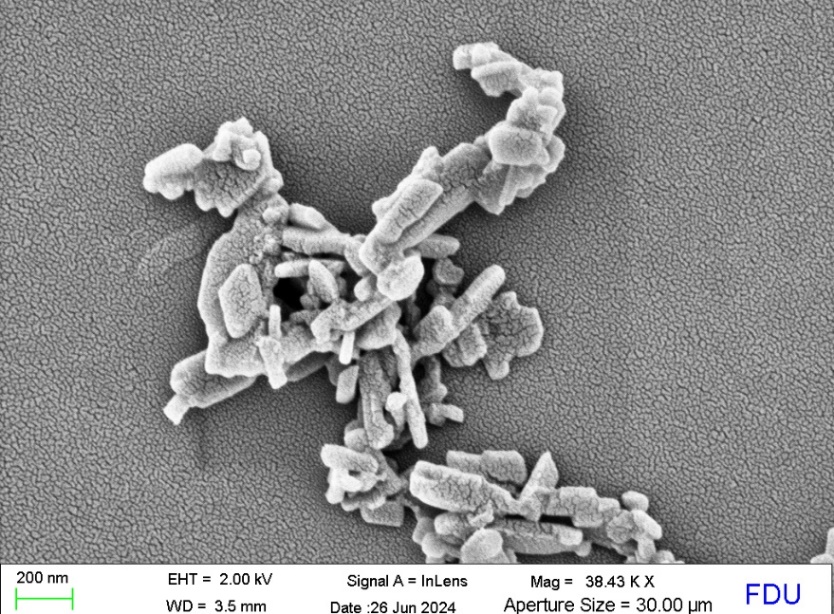


**(c)**


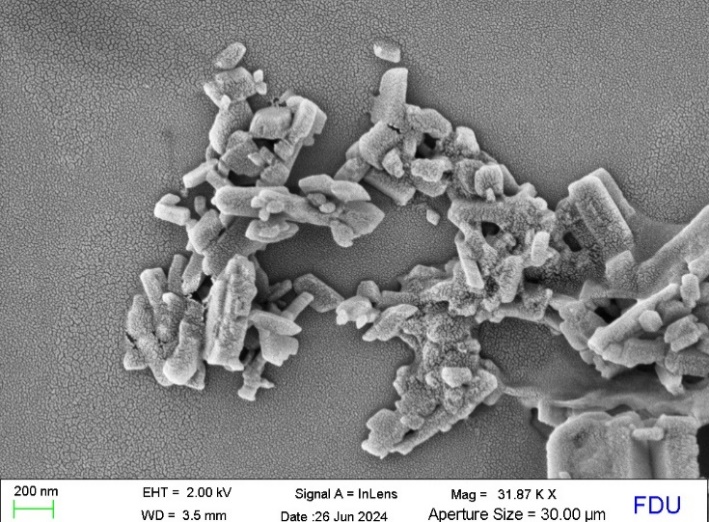


**Figure S5. SEM images of G@HOF after Na_2_S_2_O_4_ treatment (20 mM) with G@HOF for (a) 0 h, (b) 4 h and (c) 18 h. Scale bar, 200 nm**

**Figure S6. Circular dichroism spectra of free BSA and GB@HOF +Na_2_S_2_O_4_.**

**(b)**

**(a)**


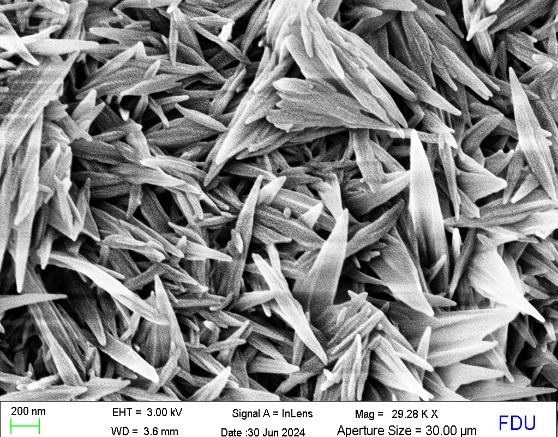

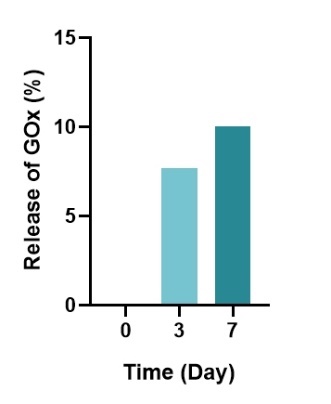


**Figure S7. (a) SEM images of G@HOF after being standed in water for 7 days, 200 nm; (b) release of GOx after G@HOF being standing in water for 7 days.**

**Figure S8. The expression of azo reductase of 4T1 cells before and after co-incubation with G@HOF for 24 h.**





**Figure S9. Bio-TEM images of G@HOF after incubation with 4T1 cells for 4 h, showing the hypoxia-responsive degradation of G@HOF**

**
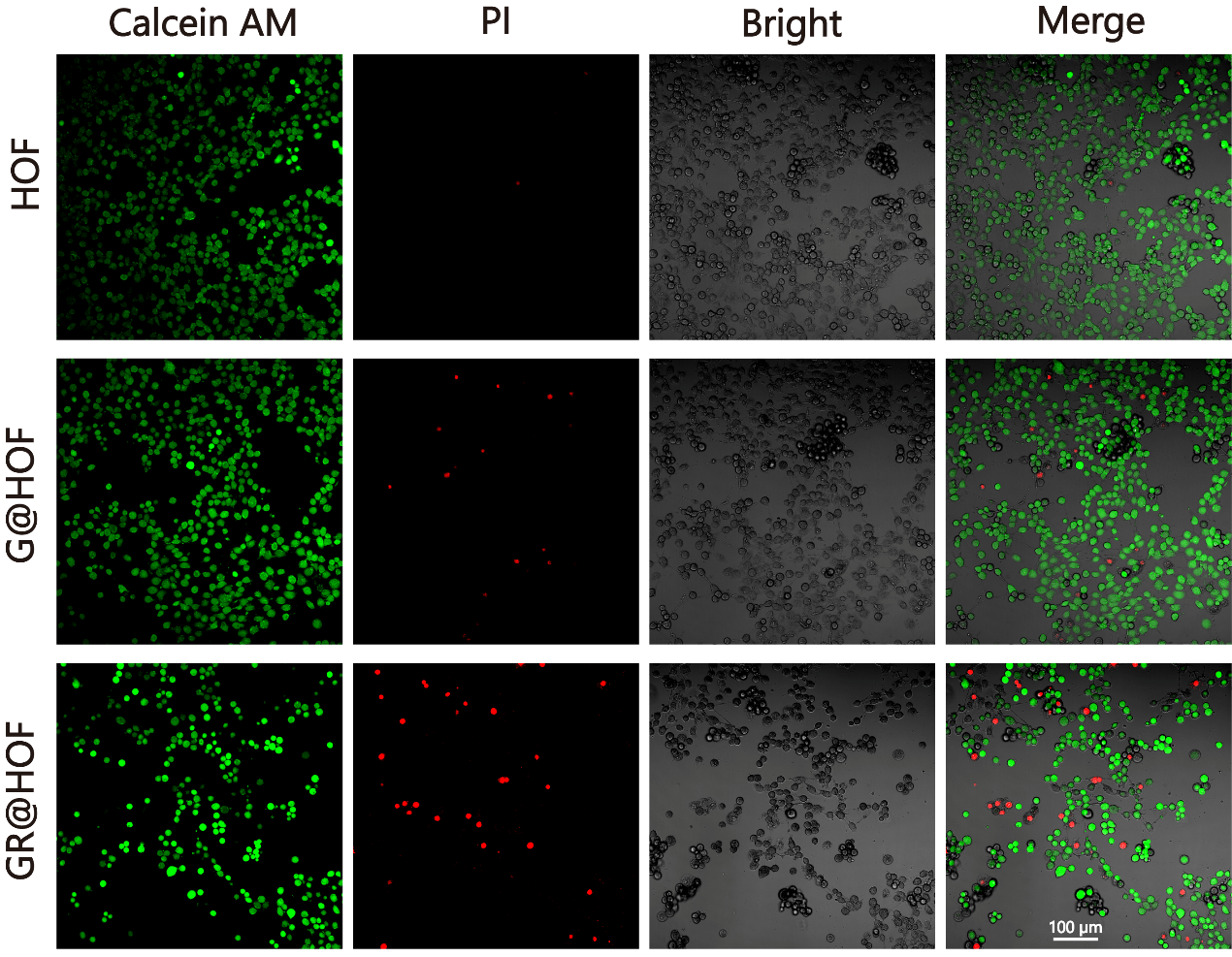
**

**Figure S10. Confocal images of 4T1 cells stained with Calcein-AM (green fluorescence, live cells) and PI (red fluorescence, dead cells) ,the scale bars are 100 μm.**

**Figure S11. Cytotoxicity studies of 4T1 cells cultivating with H_2_O_2_ (4 μM) with or without addition of GSH for 24 h.**

**Figure S12. Cytotoxicity studies of 4T1 cells cultivating with GR@HOF with low concentration of glucose (0.1 mg/mL) and additional G6P for 24 h.**

| Form of loading | Name | Loading capacity of GOx (%) | Loading efficiency of functional proteins (%) |
| --- | --- | --- | --- |
| *In-situ* capsulation | GR@HOF | 70.5 | 18.3 |
|  | GB@HOF | 71.8 | 10.5 |
|  | GC@HOF | 71.7 | 22.1 |
|  | Gβ@HOF | 44.0 | 2.9 |
| Post-absorption | G@HOF-RA | 41.7 | 1.5 |
|  | G@HOF-BSA |  | 3.2 |
|  | G@HOF-Cytc |  | 1.6 |
|  | G@HOF-βGal |  | 0.8 |

**Table S1.** **The loading efficiency of GOx and functional protein.**


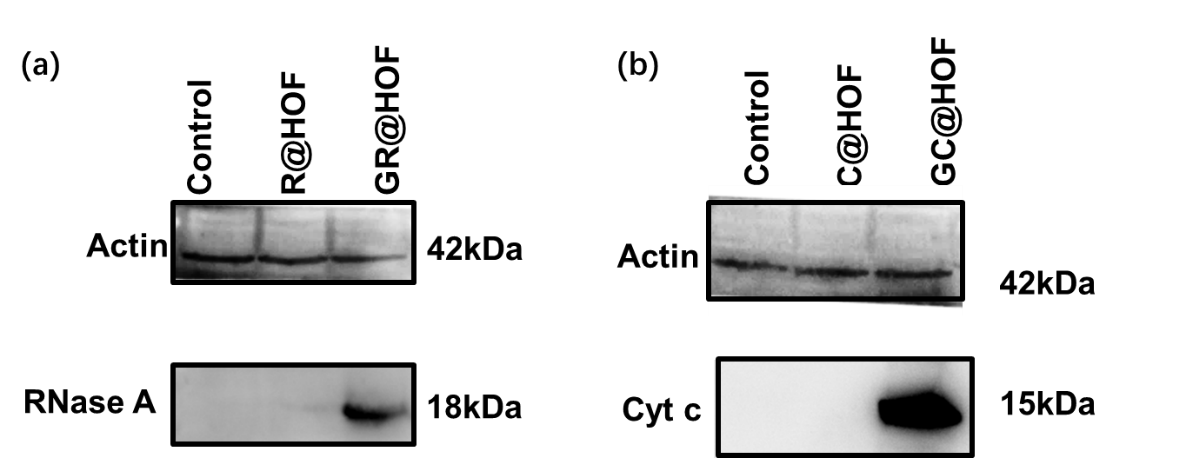


**Figure S13. Western Blot analysis of intracellular expression of RNase A with incubation of GR@HOF (a) and Cyt c with incubation of GC@HOF.**


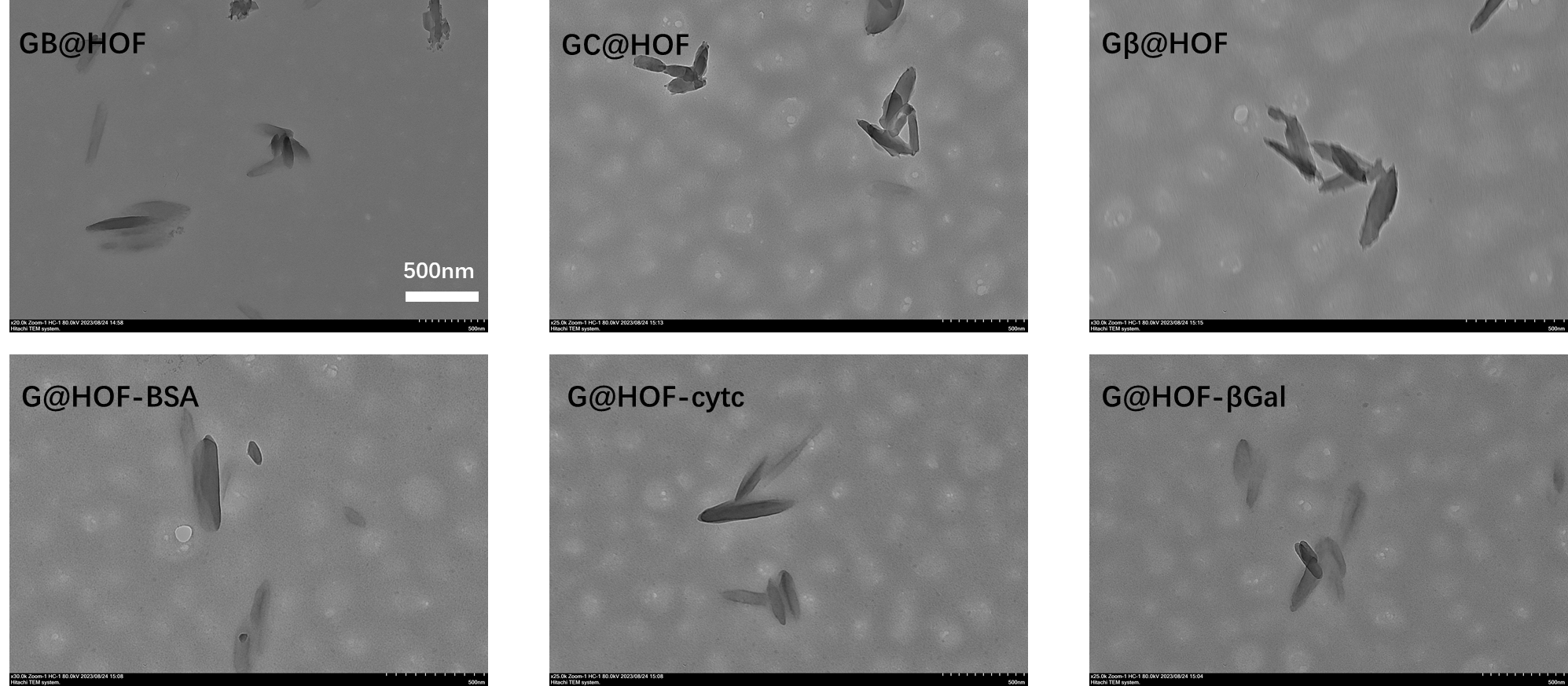


**Figure S14. TEM images of nanobiohybrid-based deliver platform with different functional proteins (BSA, Cyt c, β-gal) via in-situ encapsulation or post-adsorption.**


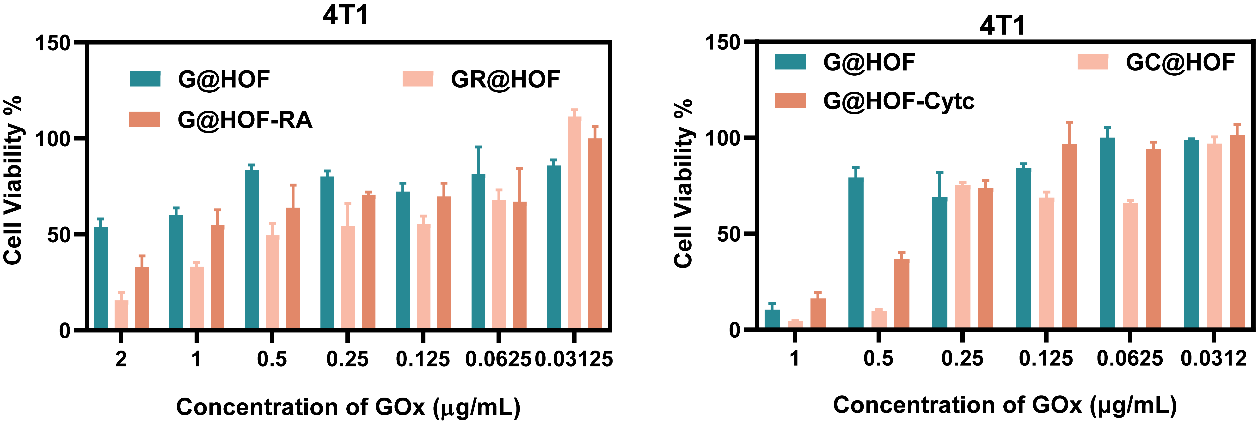


**Figure S15. Cytotoxicity studies of 4T1 cells cultivating with G@HOF-RA (left) and G@HOF-Cytc (right) for 24 h.**


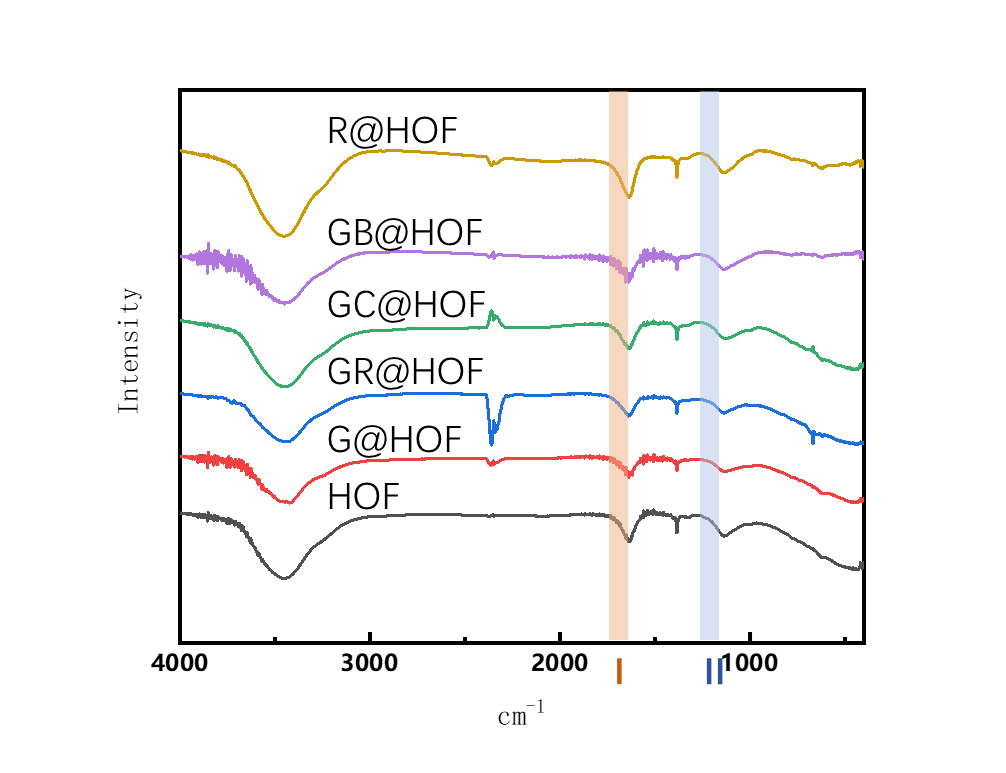


**Figure S16. FTIR spectrum of HOF, G@HOF, R@HOF, GR@HOF, Gβ@HOF and GC@HOF**


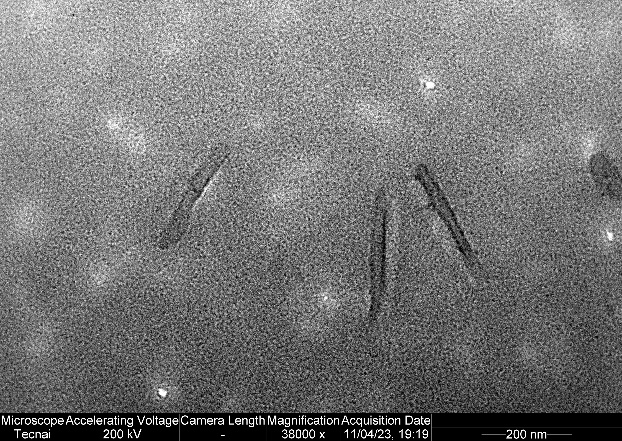

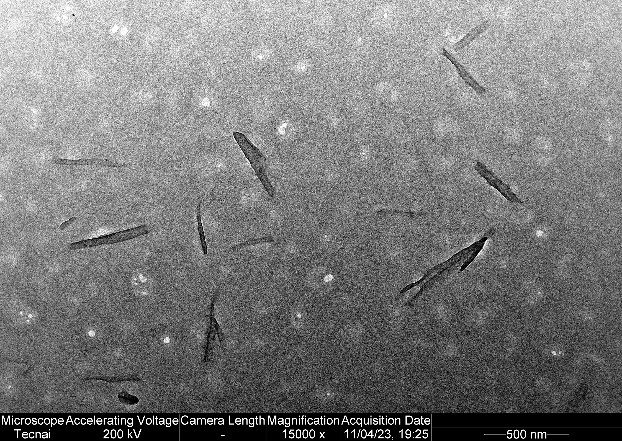


**Figure S17. TEM image of G@HOF-HA and GR@HOF-HA**


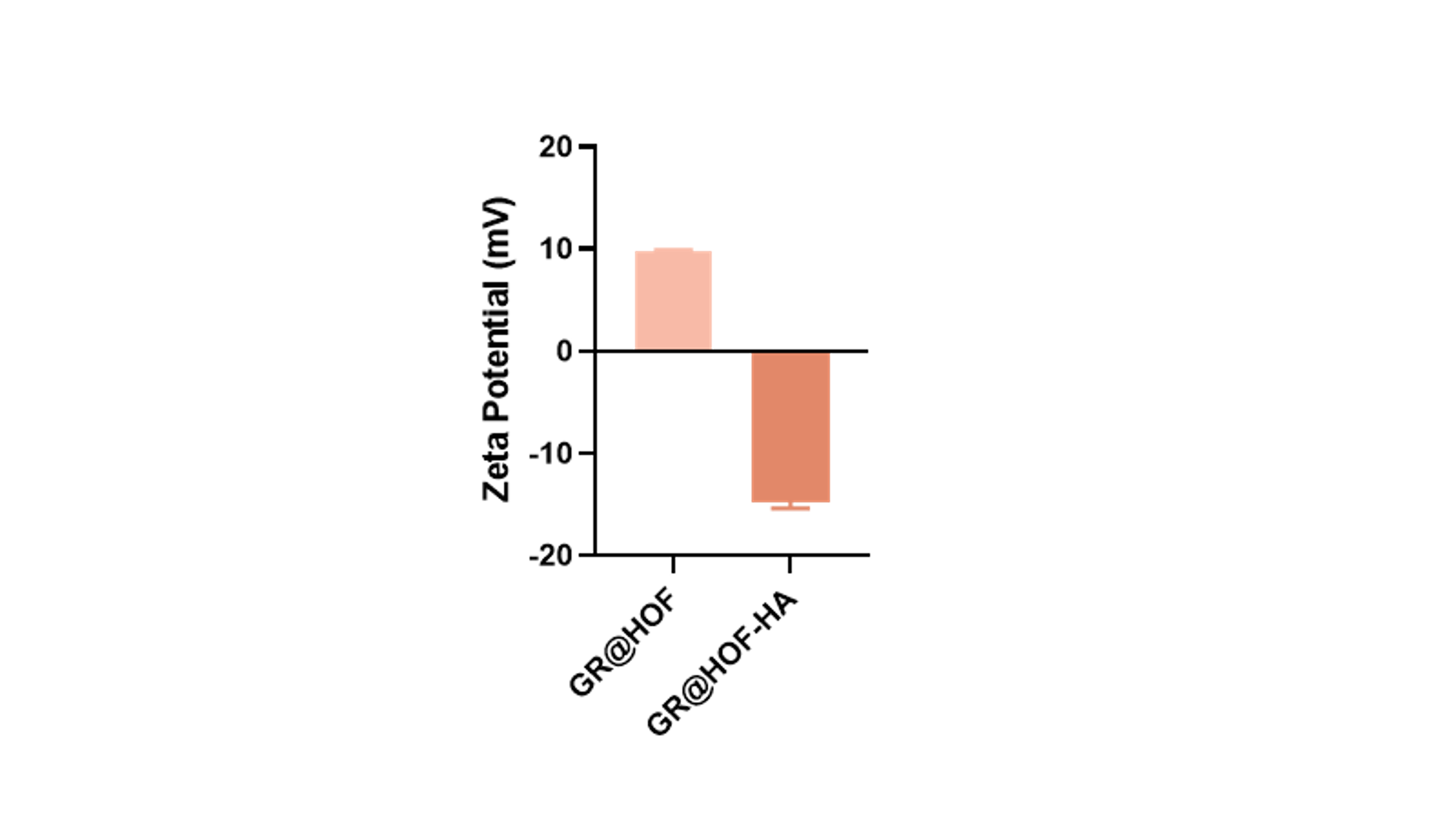


**Figure S18. Zeta potential of GR@HOF, GR@HOF-HA,**

**Figure S19. Cytotoxicity studies of HUVEC cells cultivating with G@HOF-HA and GR@HOF-HA for 24 h.**

| Parameters | No1 | No2 | No3 | Mean | SD |
| --- | --- | --- | --- | --- | --- |
| AUC_(0-∞)_^a^ | 9878.3 | 7930.5 | 9362.2 | 9057 | 582.6 |
| MRT_(0-∞)_^b^ | 6.980 | 4.765 | 6.308 | 6.018 | 0.656 |
| t_1/2_^c^ | 6.550 | 3.804 | 4.936 | 5.097 | 0.797 |

**Table S2. The pharmacokinetic parameters of GR@HOF-HA.**





**Figure S20. The pharmacokinetics of GR@HOF-HA *in vivo*.**


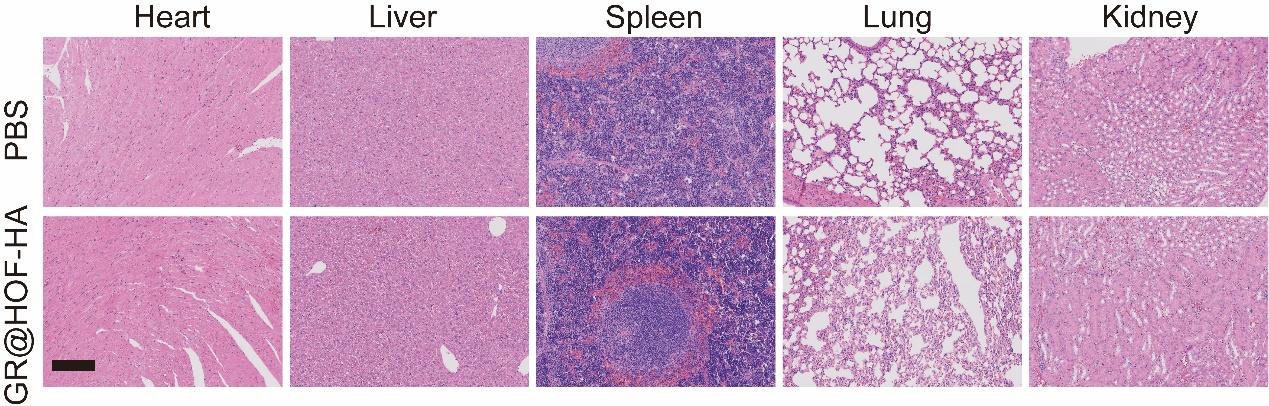


**Figure S21. H&E staining after tumor paraffin sections after the mice were treated with GR@HOF-HA or PBS. Scale bar = 100 μm.**

**Figure S22. Cytotoxicity studies of 4T1, B16F10, A549 and PC3 cells cultivating with G@HOF- and GR@HOF for 24 h.**


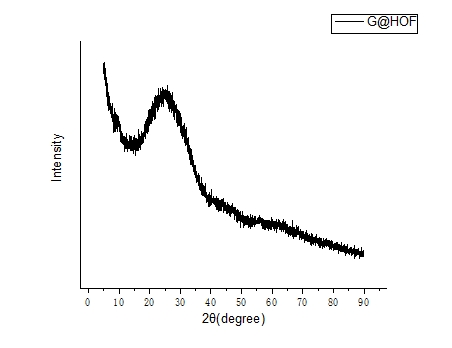


**Figure S23. The powder X-ray diffraction spectra of G@HOF.**


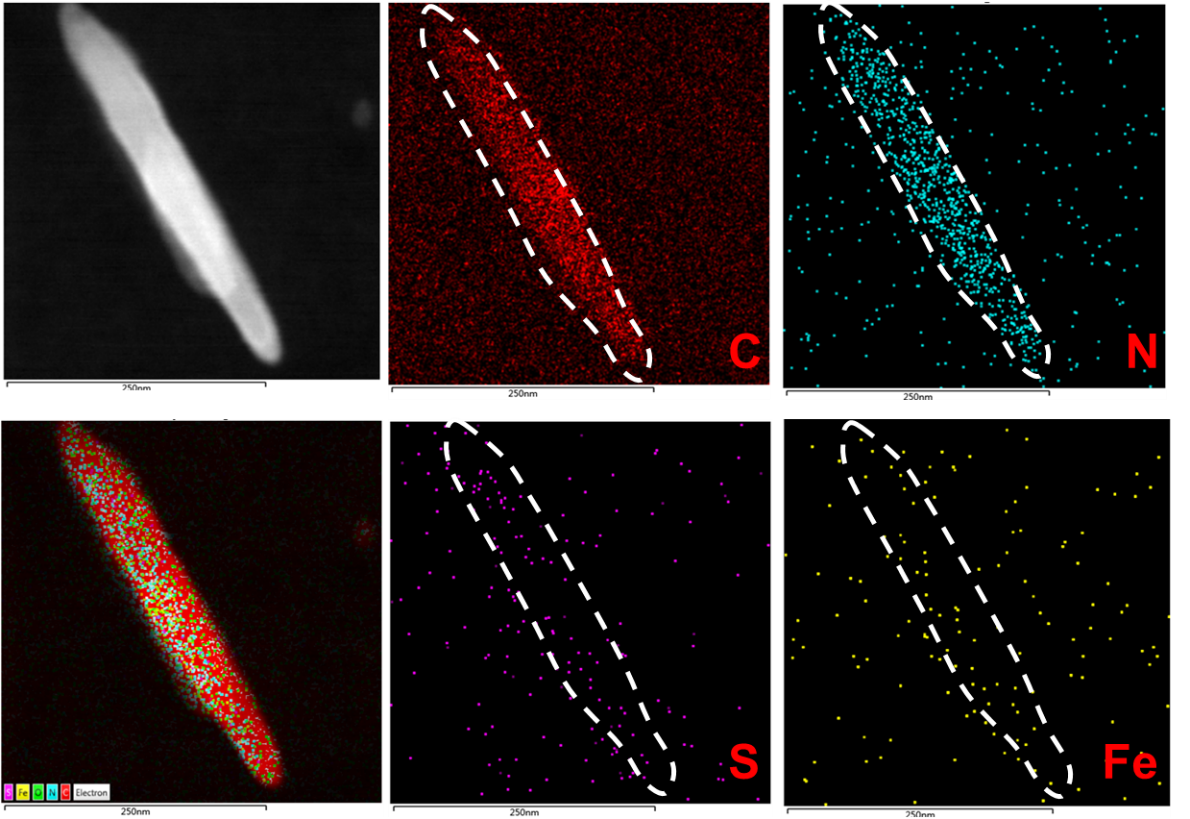


**Figure S24. EDX mapping image of GC@HOF.**
